# Management of periapical lesion with persistent exsudate

**DOI:** 10.1590/0103-6440202204818

**Published:** 2022-03-07

**Authors:** Sérgio André Quaresma, Rui Pereira da Costa, Beatriz Batalha, Maria Carlos Real Dias Quaresma, Fabiane Carneiro Lopes, Jardel Francisco Mazzi-Chaves, Antônio Ginjeira, Manoel Damião de Sousa-Neto

**Affiliations:** 1 Department of Restorative Dentistry, School of Dentistry of Ribeirão Preto, University of São Paulo, Brazil; 2 Department of Endodontics, School of Dentistry of Lisbon, Universidade de Lisboa, Lisboa, Portugal

**Keywords:** periapical cyst, decompression technique, cone beam computed tomography, persistent exsudate

## Abstract

This case describes the treatment and follow-up of a mandibular molar in an 18-year-old female with a periapical cyst. Thus, it becomes important to know which options should we take when faced with a clinical situation that we cannot resolve through conventional methods, and which techniques and approaches we have to achieve treatment success. This case showed the treatment plan and follow-up, by the use of CBCT images, from a previously treated mandibular molar with a large periapical abscess and cystic lesion, in which, the first treatment plan approach was to make the endodontic retreatment. During the chemo-mechanical preparation the presence of permanent intracanal purulent exudate made it impossible to dry the canals, impeding obturation of the root canal system. Due to this clinical situation, a surgical approach was performed with the intention of reduce this permanent exudate and to execute a decompression technique. Clinical findings, periapical radiographs and cone beam computed tomographic, indicated almost complete resolution of the radiolucency, after a one-year follow-up.

## Resumo

Este relato de caso descreve o tratamento e acompanhamento de um molar inferior em uma jovem de 18 anos com cisto periapical. Assim, torna-se importante saber quais opções devemos tomar diante de uma situação clínica que não podemos resolver pelos métodos convencionais, e quais técnicas e abordagens temos para alcançar o sucesso do tratamento. Este caso mostrou o plano de tratamento e acompanhamento, por meio de imagens de tomografia computadorizada de feixe cônico (TCFC), de um molar inferior previamente tratado com grande abscesso periapical e lesão cística, no qual, a primeira abordagem do plano de tratamento foi fazer o retratamento endodôntico. Durante o preparo químico-mecânico a presença de exsudato purulento intracanal permanente impossibilitou a secagem dos canais, impedindo a obturação do sistema de canais radiculares. Devido a essa situação clínica, foi realizada abordagem cirúrgica com a intenção de reduzir esse exsudato permanente e executar uma técnica de descompressão. Após um ano de acompanhamento, os achados clínicos e radiográficos indicaram processo de reparo.

## Introduction

Persistent infection and/or maintenance of intra- or extra-radicular microorganisms after endodontic therapy can lead to an inflammatory immune reaction of periapical tissues, leading to bone resorption as well as the development of granulomas and cysts ^1^. Cysts are divided into two groups: odontogenic and non-odontogenic cysts. Odontogenic cysts are subsequently divided into inflammatory and developmental cysts. Inflammatory cysts are further divided into radicular, residual and collateral ^1^^,^^2^^,^^3^^,^^4^^,^^5^, with a prevalence of 52.3%, being the anterior maxilla region the most often affected zone with residual cysts, followed by the lower molar region ^6^.

True cysts forms when developmental or inflammatory factors stimulate the proliferation of epithelial cells surrounding the tooth. As these cells grow, the central cells become too far from the blood vessels to receive nutrients, becoming necrotic ^1^^,^^2^^,^^3^^,^^4^^,^^7^^,^^8^. Subsequently, a cavity surrounded by epithelium is formed ^8^. The intracellular products make the cavity hypertonic, which causes fluids to enter the cavity through osmosis. In contrast, this creates hydrostatic pressure that causes bone resorption, clinical expansion, and eventually moderate pain or paresthesia ^7^. A definitive diagnosis of a true cyst can only be made with histologic examination and cannot be achieved based on clinical examination or treatment alone ^9^.

According to Nair et al. ^10^, root cysts are probably induced by the initiation of an acute inflammation focus surrounded by a delimited epithelium, leading to believe on the theory that defends that this process results from the breakdown of connective tissue by enzymatic action ^11^.

The diagnosis of cysts is a challenge for the dentist and gathering all the necessary information is essential to make a correct evaluation as well as to achieve treatment success ^1^^,^^2^^,^^3^^,^^4^.

The size of the lesion is one of the aspects that generates more doubts among the clinicians regarding what is the better treatment approach, whether surgical, non-surgical retreatment or even extraction. According to Tian et al. ^9^ factors including tooth location, a neighboring anatomic structure (sinus, nasal floor, or nerve duct), the overall health condition, and lesion size, may influence decision making for periapical surgery.

Nevertheless, Strindber et al. ^12^ and Sjogren et al. ^13^ found no significant differences in healing frequency between lesions initially larger than 5 mm and those smaller than 5 mm. In a long time period clinical study, Çaliskan ^14^ have reported 42 nonsurgically treated teeth with large cyst-like lesions. There were 73.8% of all cases completely healed with nonsurgical treatment.

The surgical decompression technique implies the creation of a small window in the cystic wall and subsequent placement of a drainage tube. Thus, the epithelial lining of the cystic lumen is confluent with the oral cavity without coalescing, causing drainage of the cystic content and equivalence between intra and extra cystic pressures ^15^^,^^16^. Techniques that reduce the cyst size so it can be later removed surgically with less associated morbidity, may be good therapeutic options ^12^, as long as there is a good acceptance and collaboration in the therapy by the patient, and mandatory periodic postoperative follow-ups ^15^^,^^16^.

The aim of this case report was to describe the management of a mandibular large lesion using a decompression technique.

## Case Report

An 18-year-old Caucasian female presented with discomfort on the right posterior region of the mandible. After informed consent was obtained, radiographic and tomographic exams were performed and a large periapical radiolucency-surrounding tooth 46 was identified. Nonsurgical root canal treatment had been performed on tooth 46, one year before. Oral examination also revealed a composite restauration on tooth 46, and a facial edema on its region. Percussion and palpation were within normal limits, and no sinus tract or mobility was detected. Periodontal probing depth was 7 mm in the mesial buccal area of tooth 46, and within normal limits on the remaining areas.

**Figure 1:**
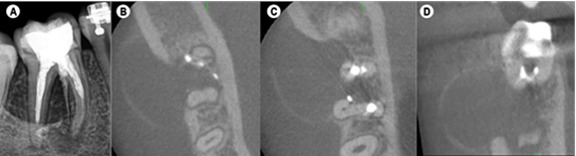
(A) Periapical radiograph with the previous treatment. (B), (C) and (D) Cross section of cone-beam computed tomographic images showing extensive lesion involving the interproximal, buccal, and lingual surfaces.

The preoperative periapical radiograph showed periapical radiolucency associated with tooth 46 (Figure 1A). Cone-beam computed tomographic (CBCT) imaging depicted a large, well-defined expansive radiolucent lesion at the apices of tooth 46, and a perforation near the mesiolingual canal (Figure 1B). The buccal cortical plate was significantly thinned and disrupted in localized areas (Figure 1B, 1C, 1D). The diagnosis was established as previously treated with asymptomatic apical periodontitis. Treatment options were discussed with the patient, including non-surgical retreatment followed by apical surgery with full enucleation and guided tissue regeneration or cystic decompression followed by non-surgical retreatment, the latter being the patient's preferred treatment option.

**Figure 2:**
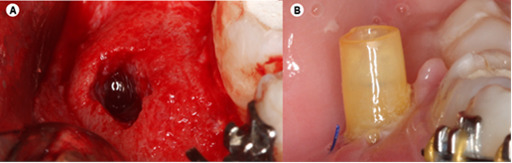
A. Flap with the hole made in the bone for drainage and tube placement. B. Decompression tube sutured to the surrounding soft tissues.

During the subsequent visit, inferior alveolar nerve block local and buccal infiltration anesthesia was performed, using 5.4 mL of 4% articaine with 1: 100.000 epinephrine (Ubistesin Forte/3M ESPE, Seefeld, Germany). An envelope incision was performed to fully visualize the area between tooth 47 and 45, thus avoiding releasing incisions close to the mental nerve emergence. A small vertical releasing incision was made distally to tooth 47 to allow flap mobility and avoid dehiscence of the flap elsewhere. Bone perforation was achieved with a small spherical bur mounted on a surgical handpiece (Figure 2A). Copious sterile saline irrigation was present throughout the surgery. A sterile venous line was custom fitted to the incised lesion wall to prevent the flanges of the incision from closing; the tube was stabilized with nylon 4-0 sutures (Figure 2B). The patient was instructed to flush both the crypt and tube with 0,2% chlorohexidine at least thrice a day for as long as the decompression was maintained. Proper antibiotic prophylaxis, anti-inflammatory and analgesic therapy was prescribed, including amoxicillin and clavulanic acid 875mg+125mg, ibuprofen 600mg and paracetamol 1g.

The patient returned one week later for post-operatory follow-up without significant complaint. The decompression tube remained unobstructed and with soft tissue margins healing adequately.

**Figure 3:**
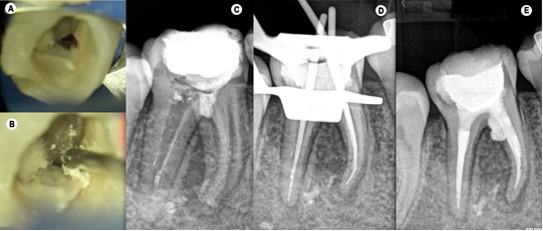
(A) Photograph showing the perforation near the mesio-lingual canal. (B) Photograph showing the placement of MTA at the perforation, using the MTA gun. (C) Immediate post-operative periapical radiograph with MTA obturation sealing the perforation. (D) Periapical radiograph with the cone-fit probe. (E) Periapical radiograph with the cone-fit probe.

In the next appointment after surgery, the patient returned for endodontic retreatment of tooth 46. Inferior alveolar nerve block anesthesia was performed with 1.8 mL of 4% articaine with 1: 100.000 epinephrine (Ubistesin Forte/3M ESPE, Seefeld, Germany) and single tooth rubber dam isolation was achieved. The root perforation near the mesiolingual canal was repaired with MTA (ProRoot; Dentsply/Tulsa Dental, Tulsa, OK, USA) (Figure 3A, 3B, 3C).

In a subsequent appointment, one week after, negotiation of the canals was performed with 10K and 15K files, and working length determined and confirmed with an electronic apex locator (Root ZX Mini, Morita Corp., Kyoto, Japan), with an irrigation regimen of 5.25% of sodium hypochlorite between each instrument. During the endodontic retreatment, to remove the filling material, the canals were shaped with ProTaper Next rotary system, until X2, with copious irrigation with 5.25% of sodium hypochlorite between files, without the use of solvent. Following preparation, each root canal was flushed using passive ultrasonic irrigation. Initially, the canals were flooded with 5 mL of 5.25% NaOCl and a 20/.01 ultrasonic insert (E1-Irrisonic, Helse Ultrasonic, Orlando, FL) was coupled to an ultrasound unit (P5 XS Bled Newtron; Satelec Acteon, Mérignac, France) and inserted 2 mm short from the working length and activated for 30 seconds at 30% power. Subsequently, the canals were flushed with 5 mL of 17% EDTA and activated for 30 seconds, followed by a new activation of 5.25% NaOCl solution for 30 seconds. A final irrigation was performed with 5 mL of distilled water ^17^. The fitting of 25.04 gutta-percha points was confirmed using periapical radiographs (Figure 3D), and root canals were dried with sterile paper points. The gutta-percha points were coated with an epoxy resin sealer (AH Plus Jet, Dentsply Sirona) and fitted slowly to full length (Figure 3E). The root canals were obturated using continuous wave of condensation technique, in combination with thermo-plasticized injectable gutta-percha backfill. After the filling, in the same session, the tooth was then restored with composite resin (Filtek Supreme XT, 3 M ESPE, Seefeld, Germany).

**Figure 4:**
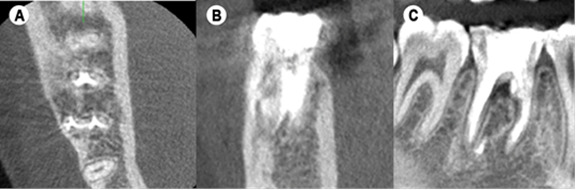
(A) Interproximal, (B) lingual and (C) buccal surfaces of one-year post-operative cone beam tomography showing almost complete resolution of radiolucent lesion.

Six weeks after the surgery, the tube was naturally expelled. The patient was recalled after 12 months (Figure 4A, 4B, 4C). Intraoral examination revealed no swelling of the soft tissues and absence of a sinus tract. Radiographs and CBCT showed an increase in density of the bone surrounding teeth 46, and an intact lamina dura (Figure 4A, 4B, 4C).

## Discussion

The management of large cystic lesions has been a subject of a continuous prolonged debate ^1^,^2^,^3^,^4^,^18^,^19^. The reported case represents a large non-resolvable cystic lesion with endodontic treatment. Lin et al. ^20^ stated that large periapical lesions and cyst like lesions may fail to heal after nonsurgical root canal treatment due to persisting intracanal or extraradicular infection, or irritants ^20^. According to the literature, in other body parts, bone lesions larger than 2,5cm are deemed critical-size lesions and have an unpredictable prognosis for complete bone regeneration ^21^^,^^22^.

Since the protocols of decompression are not consensual in the literature, the authors underline that the comfort for the patient is questionable under the use of this technique and needs to be clarified with the patient before starting the treatment. Tian et al. ^9^ showed that nowadays, there are many situations in which the 3-dimensional images produced by CBCT imaging are used to facilitate diagnosis and influence treatment in endodontics. In addition, the true size, extent, nature, and position of periapical and resorptive lesions can be assessed ^1^^,^^2^^,^^3^^,^^4^^,^^23^, and the distortion and superimposition of the 2-dimensional radiographic images can be avoided ^1^^,^^4^^,^^23^.

A cyst is a pathological cavity lined with epithelium that contains liquid, gas or other aqueous material in its content ^24^^,^^25^. These can lead to bone remodeling and consequent bone weakening, leading to functional changes and increased predisposition to infections and fractures ^26^. When the lesion is separate from the apex and with an intact, epithelial lining (apical true cyst) it could develop into a self-perpetuating entity leading to a no healing when treated nonsurgically ^1^^,^^27^^,^^28^^,^^29^.

Sometimes a large periradicular lesion may have a direct communication with the root canal system (apical pocket cyst) and respond favorably to nonsurgical treatment. Clinical studies have confirmed that simple nonsurgical treatment with proper infection control can promote healing of large lesions. When this treatment is not successful in resolving the periradicular pathosis, additional treatment options should be considered, such as marsupialization or tube decompression ^1^^,^^2^^,^^3^^,^^4^^,^^27^^,^^28^^,^^29^.

For Neaverth et al. ^30^ and Loushine et al. ^31^, the enucleation and primary closure are more congruent for cystic lesions adjacent to the nasal floor, sinus floor, mandibular nerve canal, and neighboring tooth apices, when compared with decompression ^31^^,^^32^. Liang et al. ^33^ states that full root length and pulp vitality of the adjacent teeth may be preserved with the use of surgical decompression ^33^, and the present case follows these assumptions.

Tian et al. ^9^ points several devices to achieve decompression. In this particular report was used a polyvinyl tube. This tube needs to be fixed with soft tissue suture. Although when placed in the mucosa, the suture may easily fall out ^9^, that did not happen in the reported case. The drainage period depends in most cases on the cyst size and the rate of cure and secondary enucleation is usually not necessary in the present case. In this case, it took two months for almost complete healing to take place.

The potential drawbacks of decompression include the need for long-term follow-up and patient compliance, regular cavity irrigation, unavailability of biopsies for histopathological examination, and possible infection of the exposed cavity ^9^. Gunraj ^34^ and Martin ^32^ recommend regular irrigation with saline or chlorhexidine. In this case irrigation was performed with chlorhexidine.

The end of decompression depends on radiographic image and clinical visits. Gunraj ^34^ and Martin ^32^ suggest as criteria: the end of the drainage, the radiological evidence of trabecular bone, the cavity reduction and the patient's relief. As in recent reports that compared specimens obtained from surgical decompression and from complete surgical removal of periapical cysts, the authors also did not find no recurrence of periapical cysts after decompression ^35^^,^^36^. Applying the decompression technique is more effective in larger initial lesions when carried out during more than 6 months ^35^.

The main advantages of CBCT imaging are its accessibility, easy handling and that its offers a real-size dataset with multiplanar cross-sectional and 3D reconstructions based on a single scan with a low radiation dose ^1^^,^^4^^,^^37^. Whilst Tian et al. ^9^ suggests the size of the lesion as the main factor for treatment choice. In this report, our treatment option of a surgical approach using the decompression technique prior to root canal retreatment was mainly due to the presence of persistent intracanal purulent exudate during the chemo-mechanical preparation, making impossible to dry the canals, and thus impeding obturation of the root canal system. In addition, the decompression technique promotes greater patient comfort in addition to being a minimally invasive technique compared to surgical techniques for cyst removal.

## Conclusions

This case report showed that decompression technique for treatment of periapical cysts allows a more conservative and easy approach for the clinician, and even if decompression fails, the procedure facilitates subsequent micro-apical surgery by reducing lesion size and minimizing potential damage to adjacent anatomic structures.
